# Effect of New Peripudendal Block (PPB) in the Second Stage of Labour on Perineal Relaxation and on the Reduction of Episiotomy Rate: A Randomized Control Trial

**DOI:** 10.1155/2022/9352540

**Published:** 2022-03-26

**Authors:** Artur Beke

**Affiliations:** Semmelweis University, Department of Obstetrics and Gynaecology, Budapest, Hungary

## Abstract

**Methods:**

In a prospective randomized study, we examined the extent to which the PPB we developed changed the rate of episiotomies, injury rates.

**Results:**

A total of 333 primiparas and 324 multiparas were included in the study. In the case of primiparas, we used the PPD procedure in 133 cases, while in the case of multiparas, we used it in 103 cases. The rate of episiotomy in primiparas was 89/133 (66.9%) with PPD and 181/200 (90.5%) without PPD (*p* < 0.02). In multiparas, the episiotomy rate was 30/103 (29.1%) with PPD and 140/221 (63.3%) without PPD (*p* < 0.02). In the case of primiparas, the rate of perineal injury and lesion was 33/133 (24.8%) with PPD, while without PPD it was 12/200 (6.0%). Examining the need for all surgical care (due to episiotomy and/or injury), a total of 103/133 cases of operative surgery were required with PPD (77/4%) while 183/200 cases were required without PPD (91.5%)(*p* < 0.02). In the case of multiparas, the rate of perineal injury and lesion was 11/103 (10.7%) with PPD, while without PPD it was 9/221 (4.1%). In the case of multiparas, a total of 41/103 cases required surgical care with PPD (39.8%), while without PPD, 147/221 cases required surgical care (66.5%)(*p* < 0.02).

**Conclusion:**

The PPB is simpler, requires less medication, can be easily mastered, and perineal relaxation can also be observed, reducing the need for an episiotomy.

## 1. Introduction

In the practice of labour analgesia, the pudendal nerve block (PNB) has been introduced to relieve pain in the second stage of labour [1–3]. In addition to spontaneous deliveries, it has been used in operative vaginal terminations of labour (forceps deliveries) [4–6]. For operative vaginal deliveries, comparative studies have been performed on the use of spinal analgesia and pudendal nerve block [7, 8].

The pudendal nerve block was often used in conjunction with the paracervical block and was introduced as a supplement to it [9, 10]. The paracervical block relieves the pain of the first stage of labour. The nervus pudendus block can be used in the second stage of labour [10]. During the pudendal nerve block, the local anaesthetic is injected into the area of the pudendal nerve, which contains the parasympathetic fibers originating from the vagina and perineum, thereby providing adequate analgesia in the second stage of labour. The combination of these two procedures provided adequate analgesia for both the first and second stages of labour [9, 10]. As the paracervical block and the pudendal nerve block were used together, the retreat of the paracervical block also resulted in the same reduced use of pudendal nerve block. The retreat of the paracervical block in the practice of obstetric analgesia can also be explained by the fact that the paracervically administered local anaesthetic can penetrate into the uterine arteries located here, thereby directly endangering the placenta and the intrauterine fetus.

Comparative studies have been performed to investigate the effects of epidural analgesia, pudendal nerve block, and systemic medication [11, 12]. Presumably, the proliferation of epidural analgesia also played a role in reducing the concomitant use of the traditional paracervical-pudendal block.

The use of the traditional nervus pudendus block has also been pushed into the background because it is technically more difficult to perform and requires more accurate anatomical knowledge [13]. The reason for this is that it requires a special long needle and the nerve (pudendal nerve) must be visited during the procedure. When guided from the vagina, the risk of vaginal injury increases [14]. In a conventional pudendal nerve block, a relatively larger amount (8–10 ml per side) of local anaesthetic is administered in various compositions [15–18]. The vaginal lesions experienced in vaginal births can also be explained by this [19]. In addition to higher amounts of drugs, a decrease in the pushing reflex has also been observed in some cases [16].

The aim of the study was to develop a new procedure by modifying the procedure while maintaining the beneficial analgesic and perineal relaxing effects of the traditional pudendal nerve block, the implementation of which is simpler, does not require special skills, and the method can be easily mastered. The procedure was named peripudendal block (PPB). The aim of this study was to investigate the extent to which the peripudendal block we developed reduces the rate of episiotomy in primiparous and multiparous parents during the second stage of labour.

## 2. Methods

At a prospective randomized group controlled study at the Obstetrics Clinic of Semmelweis University, we compared the effects of analgesia in the case of peripudendal block (developed by us) used in the second stage of labour and those parturients who did not receive it. In the study, we compared two groups in the experiment based on a parallel group arrangement. The studied variables have a different effect on each subject, and several layers influencing the effect are present; therefore, we found a number of cases above 100 for primiparas and multiparas, and 200 or more cases in the control group to eliminate the “biasing” effect. A computer sample size calculator was used to determine the sample size (confidence level: 95%, margin of error: 5%, population proportion: 33%). Outcomes were assessed based on data sheets completed during and after delivery. We used computerized randomization. Based on computer randomization, patients were assigned by the research nurse. Interventions for each group were communicated clearly and in sufficient detail, including how and when they were administered. Patients were included in the study after detailed information and consent.

Our work complies with the principles of the Declaration of Helsinki and is approved by the Ethical Committee of the Institution (Scientific Research Ethics Committee Clinical trial number: SE-TUKEB 33/2013, date of registration: 28^th^ March 2013). Dates of patient enrolment: 1^st^ May 2013–30^th^ April 2017 (Registry URL: https://ett.aeek.hu/tukeb/). Written informed consent was obtained from all participants.

The aim of the study was that the applied procedure not only relieves pain but also relaxes the muscles of the perineum. Therefore, we examined the rate of episiotomies and lesions in the second stage of labour to demonstrate beneficial effects.

**Figure 1:**
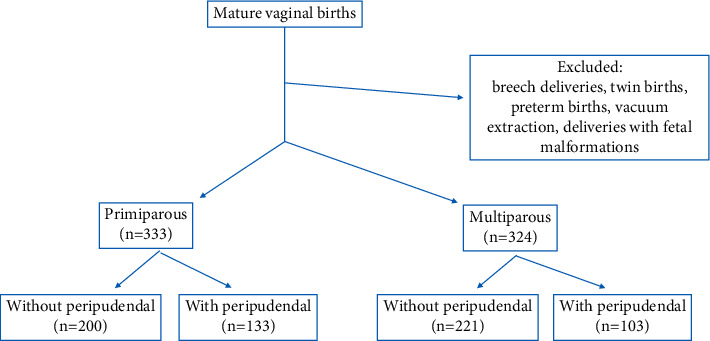
Flow diagram of the study.

The study excluded cases where episiotomy was required from the outset, such as breech deliveries, twin births, preterm births, vacuum extraction, and fetal malformations (Figure 1—Flow diagram).

In the statistical procession calculating significance, the Student-t test, Fisher's exact test, and the Chi-square test were used. In case of *p* < 0.05, the anomaly was considered as statistically significant.

### 2.1. Description of a New Method—Technical Implementation of the Peripudendal Block

**Figure 2:**
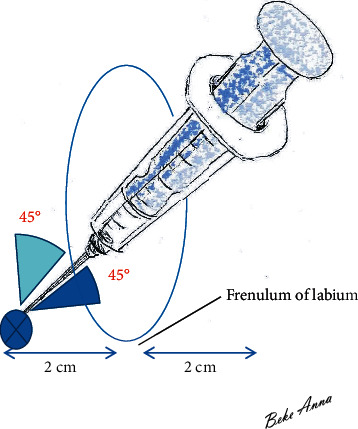
Direction of proper needle insertion. Inserting the needle at a 45-degree angle in 3D (at a 45-degree angle to both the vertical and horizontal planes—directed laterally and downward) provides the most appropriate direction.

On both sides of the perineum, the needle is guided in the direction of the spina ischiadica. Palpating the ischial spine may help determine the direction, if this is not feasible, the introduction of the needle at a 45-degree angle (both vertical and horizontal planes) provides the most appropriate direction laterally and downward (Figure 2). It does not require the use of a special needle (such as spinal needle). The needle used for conventional intramuscular injection (20–21G and 4 cm long) is suitable since it is not necessary to inject the drug deeper. Preliminary disinfection is very important.

**Figure 3:**
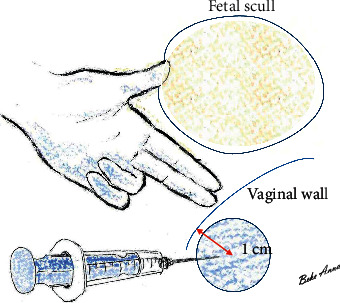
Proper protection of the skull by hand. The method is used in the second stage of labour. It is extremely important to protect the fetal skull in the cavity. Therefore the method can only be applied under the protection of the other hand. (1) Check and protect the skull with a gloved hand in the vagina. (2) Make sure the tip of the needle is about 1 cm deep from the vaginal wall—so the medicine you inject is not too superficial.

Since the method is used in the second stage of labour, it is extremely important to protect the fetal skull in the cavity. Therefore, the method should only be applied under the protection of the other hand. Insert the needle while checking with the middle and index fingers of the other “examining” hand in the vagina (Figure 3). On both sides, the same hand (if examined with the right hand: the right hand) provides protection. The examining hand not only protects the fetal skull, but it also controls the correct direction and helps prevent the needle from getting too close to the vaginal wall, endangering it (Figure 3).

**Figure 4:**
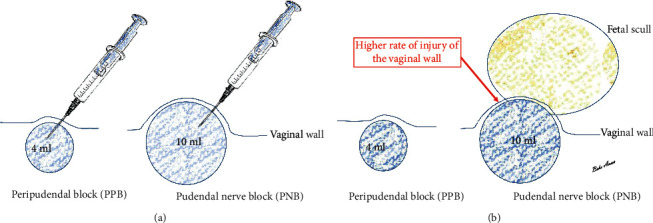
The difference between the amounts of drug administered for peripudendal block (PPB) and pudendal nerve block (PNB). (a) For peripudendal block (PPB), only 4 ml of local anaesthetic is administered per side, as opposed to the 10 ml of the drug described for conventional pudendal nerve block (PNB). (b) Due to the smaller amount of the drug, there is no risk of vaginal injury when the skull passes through. A smaller amount of local anaesthetic administered is also effective and avoids vaginal injury due to a too large (oversized) protuberance as the skull passes through the cavity.

After aspiration, when the needle has been introduced, 4 ml of 1% adrenaline-free lidocaine are administered per side. It is sufficient to inject the drug near to the pudendal nerve; there is no need to strive to reach the nerve. For peripudendal block (PPB), only 4 ml of local anaesthetic is administered per side, compared to 10 ml of the drug described for conventional pudendal nerve block (PNB). A smaller amount of local anaesthetic administered is also effective and avoids vaginal injury due to a too large (oversized) protuberance as the skull passes through the cavity (Figure 4).

## 3. Results

A total of 333 primiparas and 324 multiparas were included in the study at the 1st Department of Obstetrics and Gynaecology, Semmelweis University, who were not asked about epidural analgesia. The peripudendal block procedure was used in 133 cases for primiparas and in 103 cases for multiparas. Within two groups, those receiving and those not receiving the peripudendal block (PPB) formed a statistically comparable group (Table 1).

Based on the results, the rate of episiotomy in primiparas was 89/133 (66.9%) for peripudendal block and 181/200 (90.5%) without peripudendal block (*p* < 0.02).

In the case of multiparas, the episiotomy rate with peripudendal block was 30/103 (29.1%), while 140/221 (63.3%) without peripudendal block (*p* < 0.02).

We examined the proportion of lesions with and without peripudendal block. In the case of primiparas, the rate of perineal injury and lesion was 33/133 (24.8%) with peripudendal block, while without peripudendal block it was 12/200 (6.0%).

The higher rate was inversely proportional to the rate of episiotomies, so if no episiotomy was performed, the rate of injury was somewhat higher. Therefore, the need for all surgical care due to episiotomy and/or injury was also examined.

In this case, we obtained a more favourable result for the peripudendal block. Primiparas required surgical care in a total of 103/133 cases with peripudendal block (77/4%), while surgery was required in 183/200 cases without peripudendal block (91.5%) (*p* < 0.02).

Similar results were obtained for multiparas. In the case of multiparas, the rate of perineal injury and lesion was 11/103 (10.7%) with peripudendal block, while without peripudendal block it was 9/221 (4.1%).

In the case of multiparas, a total of 41/103 cases required surgical care with peripudendal block (39.8%), while without peripudendal block, 147/221 cases required surgical care (66.5%) (*p* < 0.02) (Table 2).

There were no complications (accidental intravenous administration), no hematoma, or bleeding at the injection site. No side effects were observed.

## 4. Discussion

Given that pain impulses, which are primarily involved in the second stage of labour, are transmitted through the pudendal nerve (S2-4), the nerve blockade is suitable for alleviating the pain of the second stage of labour. Despite the fact that many other methods are available to us today, among the regional methods, the application of the peripudendal block we have developed has a raison d'être. The reason for this lies in the fact that, in addition to pain relief, it also provides relaxation of the perineum, thereby reducing the rate of episiotomies.

Based on our study, the use of peripudendal block significantly reduced the rate of episiotomies in both primiparas (66.9% vs. 90.5%) and multiparas (29.1% vs. 63.3%). Reducing the rate of episiotomies also had an impact on the rate of surgical care. There was a significant decrease in the rate of surgical care for both primiparas (77.4% vs. 91.5%) and multiparas (39.8% vs. 66.5%).

Based on our studies, although the rate of injuries (perineal injury and lesion) increased with the use of PPD, it was probably due to the fact that episiotomy was required less frequently due to perineal relaxation. Abandonment of the episiotomy increased the incidence of injuries.

Therefore, we examined the need for all surgical care, i.e., suturing the episiotomy and suturing the lesions together. We found that with PPD, the need for surgical care was lower for both primiparas and multiparas. So overall, we obtained favourable results for surgical care with PPD.

In addition to the beneficial effects, the new method we have introduced can be used to draw attention to a number of aspects. These include the amount of drug administered, the anaesthesia of the episiotomy, and the combination of methods.

### 4.1. Amount of Drug Administered

An important issue is the amount of medication administered. The volume mentioned in literature data in the case of the traditional pudendal nerve block (8–10 ml of local anaesthetic) can no longer be ignored during the second stage of labour. This volume reaches the limit that we have to consider, in addition to the circumference of the fetal head descending down the cavity. A large amount of medicine can already be a “barrier”. Of course, this obstruction does not slow down the childbirth; however, the downward movement of the fetal head is more likely to cause vaginal injury due to the loosening tissues after the administration of the drug. According to our observations, 4 ml of local anaesthetic per side is sufficient to achieve an adequate effect, but it does not increase the number of vaginal injuries (Table 3).

### 4.2. Anaesthesia of Episiotomy

If a peripudendal block was used in the second stage of labour and an episiotomy was needed, the peripudendal block alone is sufficient for the analgesia of the episiotomy. No additional perineal infiltration is required beforehand. However, it is advisable to supplement the anaesthesia with perineal infiltration before the perineal repair following delivery. After the birth, it is recommended to start with a peripudendal block prior to suturing the incision of the episiotomy (central: on both sides and mediolateral: on the same side), followed by perineal infiltration. If this sequence is followed, the perineal infiltration will not be painful for the parturient (Table 4).

### 4.3. Combination of Methods

Different analgesic procedures can be used in conjunction with each other. Despite the fact that during epidural analgesia, the diffusing local anaesthetic reaches even the S2-4 segment, therefore also blocking the innervation area of the pudendal nerve and relaxing the perineum. However, this effect is not fully realized in all cases. Assessing the perineal relaxation—the extent to which the perineum has relaxed (loosened), whether the perineum is expected to tear, whether an episiotomy is needed—can only be performed in the second stage of labour. Therefore, epidural supplementary dosage is not available in most cases. In such cases, in addition to the existing epidural analgesia, peripudendal block may be used as a supplement.

## 5. Conclusion

With the widespread use of humanized, family-centred obstetrics, there is a growing need to provide the right conditions during labour and delivery.

The traditional pudendal nerve block (PNB) was previously introduced in the practice of labour analgesia as an adjunct to the paracervical block; however, epidural analgesia displaced both. The traditional pudendal nerve block, in which the local anaesthetic is administered around the parasympathetic fibers of the pudendal nerve originating from the vagina and the perineum, thereby providing adequate pain relief in the second stage of labour. However, the procedure is complicated, requires a special needle, and can only be performed with proper training. The study is limited by the fact that proper practice and training are required to apply the method and can only be done in the second stage of delivery. The limitation of the study is that it is a new method, and therefore proper reproducibility requires the acquisition of the method by other researchers. Internal validity of the study: the causal relationship of the study is reliable as it is not influenced by other factors or variables. External validity of the study: the results of the study can be applied and generalized to other births and groups.

The new procedure we have developed, the peripudendal block (PPB), is simpler, requires less medication, and can be easily mastered in routine obstetric pain relief practice. During its use, in addition to analgesia, the relaxation of the perineum can also be observed, reducing the need for an episiotomy. It can be combined with other methods such as nitrous oxide or epidural analgesia. Its advantage is that it provides adequate pain relief for both episiotomy and perineal suturing.

The relaxation of the perineum makes the second stage of labour more gentle, reduces the need for an episiotomy, and reduces the possibility of perineal injury. Perineal relaxation may be particularly important in preterm births, where in addition to the weaker, less calcified skull, the more vulnerable subependymal vessels are also at increased risk.

## Figures and Tables

**Table 1:** Statistical comparison of demographic and clinical data of the studied groups.

	Primiparous			Multiparous		
	Without peripudendal	With peripudendal		Without peripudendal	With peripudendal	
Age (average) ± SD	27.08 ± 4.72	27.30 ± 4.90	NS	30.37 ± 4.28	31.27 ± 3.98	NS
Weight (average) ± SD (kg)	65.62 ± 10.59	66.12 ± 10.63	NS	62.38 ± 8.61	64.58 ± 9.59	NS
Height (average) ± SD (m)	1.67 ± 0.08	1.67 ± 0.07	NS	1.67 ± 0.06	1.67 ± 0.07	NS
BMI (average) ± SD	23.52 ± 3.56	23.67 ± 3.60	NS	22.45 ± 2.92	23.22 ± 3.36	NS
Pregnancy week (average) ± SD	39.12 ± 1.14	39.08 ± 1.07	NS	38.92 ± 1.15	38.95 ± 1.18	NS
Fetal weight (average) ± SD	3360.75 ± 400.64	3286.09 ± 456.22	NS	3431.76 ± 396.26	3461.65 ± 445.15	NS
Oxytocin infusion	115 (57.5%)	70 (52.6%)		92 (41.6%)	42 (40.8%)	
Meconium	21 (10.5%)	9 (6.8%)		23 (10.4%)	8 (7.6%)	

**Table 2:** Rate of episiotomy and perineal and/or vaginal injuries depending on whether peripudendal block was used or not.

	Primiparous					Multiparous				
	Without peripudendal		Peripudendal		*p*	Without peripudendal		Peripudendal		*p*
	*n*	%	*n*	%		*n*	%	*n*	%	
**With episiotomy**	**181**	**90.5**	**89**	**66.9**	*p* < 0.02	**140**	**63.3**	**30**	**29.1**	*p* < 0.02
Without injury	171	85.5	70	52.6		138	62.4	30	29.1	
With injury	10	5.0	19	14.3		2	0.9	0	0.0	
**No episiotomy**	**19**	**9.5**	**44**	**33.1**		**81**	**36.7**	**73**	**70.9**	
Without injury	17	8.5	30	22.6		74	33.5	62	60.2	
With injury	2	1.0	14	10.5		7	3.2	11	10.7	
**Total**	**200**	**100.0**	**133**	**100.0**		**221**	**100.0**	**103**	**100.0**	
Without injury	188	94.0	100	75.2		212	95.9	92	89.3	
With injury	12	6.0	33	24.8		9	4.1	11	10.7	
Operative suture was necessary because of episiotomy or injury	**183**	**91.5**	**103**	**77.4**	*p* < 0.02	**147**	**66.5**	**41**	**39.8**	*p* < 0.02

**Table 3:** Comparison of the peripudendal block with the traditional pudendal nerve block.

Peripudendal block (PPB)	Pudendal nerve block (PNB)
Transperineal administration It does not increase the risk of vaginal lesions It does not require any special skills It does not require a special needle/normal needle length/4 ml of drug per side	Transperineal or transvaginal administration The transvaginal administration increases the risk of vaginal lesions It requires special skills It requires a special needle/long needle/8–10 ml of drug per side

**Table 4:** Comparison of the peripudendal block with the perineal infiltration.

Peripudendal block (PPB)	Local analgesia
It can be implemented earlier There is time for the effect to develop Episiotomy does not hurt It relaxes the perineum It also anaesthetizes the suturing of the perineum It can be supplemented with local analgesia	When the fetal skull stretches the perineum No adequate effect is obtained Episiotomy may be felt by the mother It only relieves pain, no perineal relaxant effect Additional infiltration needed for suturing the perineum

## Data Availability

The data used to support the findings of this study are available from the corresponding author upon request.
